# The Phantom of the PCR: detection and consequences of spurious UMIs in mainstream RNA sequencing

**DOI:** 10.64898/2026.08.01.742199

**Published:** 2026-08-06

**Authors:** Ken Sugino, Tzumin Lee

## Abstract

Unique molecular identifiers (UMIs) support digital molecular counting by tagging molecules before amplification, but assume that UMIs are incorporated only during reverse transcription. Residual UMI-bearing oligonucleotides can instead reprime during preamplification PCR, creating “phantom” UMIs on genuine cDNA that inflate counts and evade standard deduplication. We model phantom generation as a two-state branching process and show that it produces a heavy-tailed reads-per-UMI distribution distinct from that of true UMIs. Using this signature, PhantomUMI detects and estimates contamination from clone-size distributions, subject to a coverage-dependent identifiability limit. Across 23 datasets spanning published studies and companion experiments, we find signatures consistent with phantom-UMI generation, including in current 10x GEM-X chemistry. Simulations show that phantoms inflate molecule counts and distort fold-changes. Model-based correction removes average count inflation but does not recover the distorted fold-changes, indicating that phantom UMIs are best prevented experimentally, as implemented in the companion Omega-seq method.

## Full Text Availability

The license terms selected by the author(s) for this preprint version do not permit archiving in PMC. The full text is available from the preprint server.

